# Role of Cys73 in the thermostability of farnesyl diphosphate synthase from *Geobacillus stearothermophilus*

**DOI:** 10.1007/s13205-017-0792-8

**Published:** 2017-07-10

**Authors:** Petrus Yesaya Samori, Koki Makabe, Norimasa Ohya, Bunpei Hatano, Satoshi Murakami, Tatsuro Kijima

**Affiliations:** 1 Department of Biochemical Engineering, Graduate School of Science and Engineering, Yamagata University, 4-3-16 Jonan, Yonezawa, Yamagata 992-8510 Japan; 2 Department of Material and Biological Chemistry, Faculty of Science, Yamagata University, 1-4-12 Kojirakawa, Yamagata, Yamagata 990-8560 Japan

**Keywords:** Farnesyl diphosphate synthase, *Geobacillus stearothermophilus*, Thermostability, Crystal structure, Thermal unfolding simulation

## Abstract

Farnesyl diphosphate synthase (FPPase) is an enzyme that catalyzes the condensation between one molecule of dimethylallyl diphosphate (DMAPP) and two molecules of isopentenyl diphosphate (IPP) to produce farnesyl diphosphate (FPP). FPP is an important precursor in the isoprenoid synthesis pathway. In this study, the crystal structure of FPPase from *Geobacillus stearothermophilus* (*Gs*FPPase) was determined at 2.31 Å resolution. The structure of *Gs*FPPase shows a three-layered all α-helical fold and conserved functional domains similar to other prenyltransferases. We have analyzed the structural features of *Gs*FPPase related to thermostability and compared it with those of human and avian mesophilic FPPases. “Semi-conserved” regions which appear to be possible features contributing to the thermostability of FPPase were found.

## Introduction

Isoprenoids are a highly diverse group of compounds which play important roles in the physiological processes of all free-living organisms. The fundamental step in the biosynthesis of isoprenoids is the chain elongation of prenyl diphosphate precursors by prenyltransferases. These enzymes catalyze the successive condensation between isopentenyl diphosphate (IPP, C5) and allylic diphosphates to produce prenyl diphosphates with various chain lengths and stereochemistries, thus, they are commonly classified based on the chain length and the geometry of the double bond of the final product that is formed by the reaction (Ogura and Koyama 1998).

Farnesyl diphosphate synthase (FPPase; EC 2.5.1.10) is one of the prenyltransferases and occupies a central branch point in the prenyl chain elongation pathway. It catalyzes the condensations of IPP with dimethylallyl diphosphate (DMAPP, C5) and with geranyl diphosphate (GPP, C10) to give the key precursor (*2E*, *6E*)-farnesyl diphosphate (FPP, C15) as the final product (Eberhardt and Rilling 1975).

Because of its critical role in the pathway, FPPase has been used in both mechanistic and synthetic studies. Poulter and Rilling used FPPase from avian liver and porcine liver to study the mechanism of chain elongation (Poulter and Rilling 1978). Koyama et al. used porcine liver FPPase to develop a novel chiral synthesis method and successfully applied the method to the chiral synthesis of faranal and 4-methyl-juvenile hormone (Koyama et al. 1980; Kobayashi et al. 1980; Koyama et al. 1987). Also, Maki et al. showed that porcine liver FPPase can be used to synthesize the butterfly hair-pencil pheromone (Maki et al. 1995). These studies have demonstrated that FPPase has potential applications in the synthesis of a great variety of organic compounds. However, the practical uses of FPPase in industrial applications are limited due to the instability of the enzyme and the difficulty in obtaining it in significant amounts.

Thermostable FPPase is becoming an increasing necessity to provide an enzyme which is more stable and more compatible with industrial application. Genes encoding thermostable FPPases have been isolated and prepared from some thermophilic bacteria. For example, bifunctional enzyme farnesyl diphosphate/geranylgeranyl diphosphate synthase and the gene encoding it were isolated from high thermophile *Methanobacterium thermoauototrophicum*, and the enzyme showed a high thermal stability (Chen and Poulter 1993). A gene encoding thermophilic FPPase was also isolated from medium thermophile *Geobacillus stearothermophilus* (*Bacillus stearothermophilus*) and was efficiently overexpressed in *Escherichia coli* host cells (Koyama et al. 1993).

*Geobacillus stearothermophilus* FPP synthase (*Gs*FPPase) has been shown to display thermal stability properties. The enzyme is not deactivated even after heat treatment at 65 °C for 100 min. Although many years have passed since studies of *Gs*FPPase began, the factors that determine its thermostable properties still remain unclear.

In their studies on the roles of cysteine residues in *Gs*FPPase, Koyama et al. found that replacing Cys residue at position 73 with Phe caused the enzyme to become very sensitive to heat treatment (Koyama et al. 1994). Here, we verified the effect of Cys73 on the thermostability of *Gs*FPPase by solving its crystal structure at a resolution of 2.31 Å and conducted thermal unfolding simulations.

## Materials and methods

### Expression and purification

Wild-type *Gs*FPPase was overexpressed and purified according to the method reported by Koyama et al. (1993), with some modifications. *E. coli* cells harboring expression plasmid that carry wild-type *Gs*FPPase gene were cultured overnight in Luria Broth (LB) medium containing 100 μg/ml of ampicillin at 37 °C. Cultured cells were transferred to 100 volumes of the same fresh medium and were grown further at 37 °C to an approximate OD_600_ value of 0.6. Isopropyl β-d-1- thiogalactopyranoside (IPTG) was then added to a final concentration of 0.1 mM and the solution was incubated overnight. The cells were harvested and disrupted by sonication in 10 mM Tris–HCl buffer (pH 8.0). The homogenate was heated at 55 °C for 60 min, fractionated with 35–60% ammonium sulfate solution, and purified using an anion exchange chromatography column DEAE-TOYOPEARL (Tosoh Corporation, Japan).

### Crystallization and data collection

The purified protein sample was dialyzed against 10 mM Tris–HCl buffer (pH 7.0) and concentrated to 14.5 mg/mL using Amicon Ultra concentrator (MWCO 10,000 Da; Millipore, USA). Initial crystal screening was performed by the sitting-drop vapor diffusion method using Crystal Screens I and II (Hampton Research, USA) and JCSG+ (Qiagen, USA) sparse matrix screening kit. Optimization was performed using the hanging drop vapor-diffusion method, where a drop initially contained 1 μL each of the reservoir solution and purified protein sample. Crystals for data collection were obtained using a reservoir solution consisting of 0.1 M Tris–HCl (pH 8.5), 34% PEG400, and 0.1 M LiCl at 37 °C.

### Structure determination

Crystals of *Gs*FPPase were frozen in liquid nitrogen using the crystallization solution as a cryoprotectant (34% PEG400). The X-ray diffraction data were collected at the BL-5A beamline (KEK Photon Factory, Tsukuba, Japan). The diffraction images were processed with HKL2000 (Otwinowski and Minor 1997) and SCALA of the CCP4 program suite (CCP4 1994). The structure of *Gs*FPPase was determined by molecular replacement using the program MOLREP in CCP4 using FPP synthase structure from *Staphylococcus aureus* (PDB ID 1RTR) as a search model. In the further refinement, 5% randomly chosen reflections were set aside for calculating R-free. Refinement was carried out with Refmac5 in CCP4. The model building was carried out using the Coot program. The coordinates have been deposited in the PDB with entry code 5AYP. Molecular graphics were generated using 5AYP. The data collection and refinement statistics are summarized in Table 1.

**Table 1:** Data collection statistics

Data collection	*Geobacillus stearothermophilus* FPPase
Wavelength (Å)	1.000
Resolution range (Å)	20.00–2.31
Space group	P2_1_2_1_2_1_
Unit cell	*a* = 49.02 Å, *b* = 95.16 Å, *c* = 103.35 Å *α* = 90.00°, *β* = 90.00°, *γ* = 90.00°
No. of unique reflections	20,709
Completeness (%)	99.34
Average *I*/*σ*(*I*)	19.3
*R*_work_/*R*_free_	0.211/0.252
RMS bond lengths (Å)	0.011
RMS bond angles (°)	1.432
Mean B value (Å^2^)	49.194
No. of atoms	4029
Water molecules	32
Ramachandran favored (%)	95
Ramachandran allowed (%)	3
Ramachandran outliers (%)	2

### Melting point analysis

The thermal stability of *Gs*FPPase was measured using differential scanning calorimetry (DSC; VP-capillary DSC, GE healthcare, USA). A protein sample with 1 mg/mL in a 50 mM phosphate (pH 7.0) and 100 mM NaCl buffer was used. Scanning speed was 60 °C/1 h.

### Missing loops modeling, point mutation modeling, and thermal unfolding simulation

Insertion of the missing loops of *Gs*FPPase was carried out using the ab initio method implemented in Modeller 9.16 (Fiser et al. 2000). The quality of each model was validated using ProSa (Wiederstein and Sippl 2007) and SAVES server (Laskowski et al. 1996). The resulting structure with the best quality was then used to construct the point-mutated enzyme C73F structure using Modeller 9.16. The C73F model was minimized further with the steepest descent algorithm followed by the conjugate gradient algorithm by USFC Chimera (Pettersen et al. 2004), which was developed by the Resource for Biocomputing, Visualization, and Informatics at the University of California, San Francisco. Thermal unfolding simulations were performed using CNA web server (http://cpclab.uni-duesseldorf.de/cna/) (Krüger et al. 2013). CNA web server is a web interface for performing rigidity theory-based thermal unfolding simulations based on Constrain Network Analysis (CNA) approach (Pfleger et al. 2013a). CNA models a protein as a constraint network, and by continuously removing non-covalent constraints (i.e., hydrogen bonds) from the network, it simulates the thermal unfolding process of the protein.

In this study, the simulation performed by CNA was carried out with the ‘ensemble of network-single structure’ analysis type for each enzyme, number of network topologies in each ensemble was set to 50. The network ensembles are generated from a single input structure using fuzzy-constraint definitions implemented in CNA. For the simulation parameters, E-cutoff for hydrogen bonds was set to default and hydrophobic cutoff was treated in a temperature-dependent manner. More specifically, by decreasing the E-cutoff for hydrogen bonds from an initial value of −0.1 to −6.0 kcal mol^−1^ with a step size of 0.1 kcal mol^−1^, hydrogen bonds were removed from the network in the bond strength order, based on the idea that stronger hydrogen bonds break at higher temperature. On the other hand, more hydrophobic tethers were added to the network by increasing the hydrophobic cutoff from an initial value of 0.25 Å to a terminal value of 0.40 Å, based on the fact that strength of hydrophobic interactions increases with increasing temperature (Schellman 1997).

CNA performs rigidity analysis of a protein by computing global and local indices. Global indices are used to identify phase transition points *T*_p_, where the network shifts from rigid to flexible, while local indices are used to characterize the flexibility of each bond in the network. In the case of global indices, here we only consider the cluster configuration entropy *H*_type2_, because *T*_p_ which are calculated using this modified H have been found to be related to the thermostability of proteins, i.e., the melting temperatures (*T*_m_) of proteins (Radestock and Gohlke 2008, 2011; Pfleger et al. 2013b).

## Results and discussion

### Overall structure of *Gs*FPPase

**Fig. 1:**
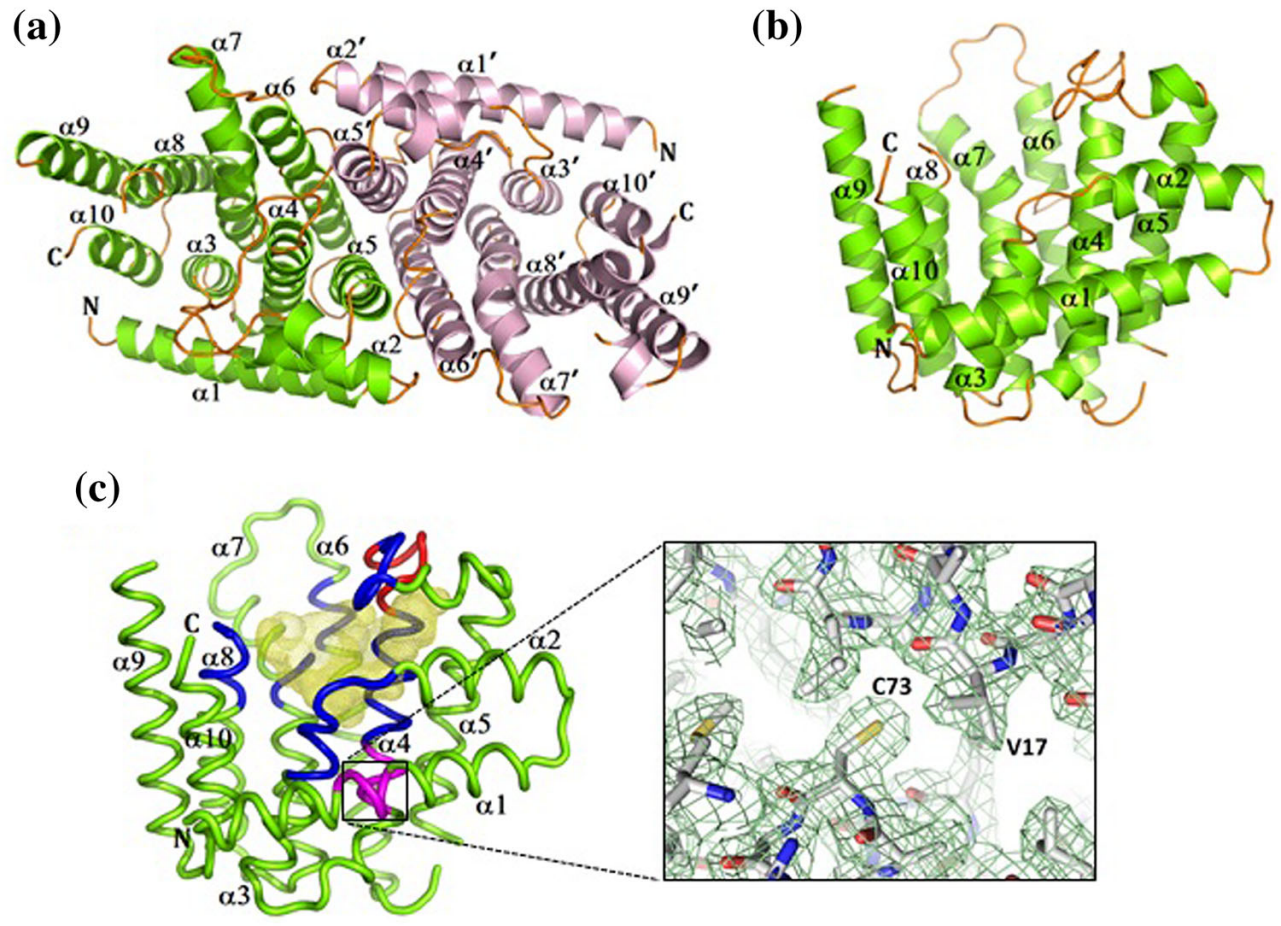
Overall structure of *Gs*FPPase. **a** Cartoon representation of *Gs*FPPase homodimer. Chain A and chain B are shown in* light green* and *pink*, respectively. Connecting loops are shown in *orange*. **b** Monomeric structure of *Gs*FPPase, with α-helices, *N*-terminal, and *C*-terminal are marked correspondingly. **c** Tube representation of *Gs*FPPase monomer. Five conserved regions that form the wall of the central cavity (*yellow mesh*) and the first aspartate-rich motif (FARM) are shown in *blue* and *red*, respectively. Two semi-conserved regions are shown in magenta. Cys73 (inset, *lime green mesh* represent 2Fo-Fc electron density map contoured at 1.0σ) is located on the semi-conserved region of helix α4

The apo form of *Gs*FPPase was crystallized in a *P2*_*1*_ *2*_*1*_ *2*_*1*_ space group with one homodimer in the asymmetric unit. *Gs*FPPase homodimer is stabilized by hydrophobic interactions involving helices α5 and α6 from both monomers. The dimer interface buries 3350 Å^2^ of accessible surface area per monomer (Fig. 1a). Because *Gs*FPPase is active as a homodimer (Koyama et al. 2000), it is reasonable to consider that the dimer occurs in solution has a similar orientation with molecules in the crystal lattice shown in Fig. 1a.

Like FPPases from other species, each monomer of *Gs*FPPase exhibits a three-layered all α-helical prenyltransferase fold. The typical fold consists of ten α-helices and connecting loops, with the first, second, and third layer formed by helices α1 and α2, helices α3–α5 and α10, and helices α6–α9, respectively (Fig. 1b). A large central cavity located within the bundle of these α-helices is the putative active site. The wall of this cavity contains five amino acid sequences that are highly conserved among prenyltransferases (Fig. 1c). Conserved first aspartate-rich motif [FARM, DDXX (XX) D: residues 86–92], was found in helix α4, located on the surface of the cavity. By contrast, second aspartate-rich motif (SARM, DDXXD: residues 224–228), which is supposed to exist in α8 on the opposite wall from FARM, could not be traced in the electron density map. This is because SARM is located near a flexible loop connecting α8 and α9 (residues 229–256), whose flexibility caused the electron density of SARM and the loop itself to become invisible in the apo-*Gs*FPPase structure. There is also a small missing loop between α4 and α5 containing residues 130 and 131. Cys73 is a part of a “semi-conserved” region and is located in helix α4, exactly on the intersection point of orthogonally positioned α1 and α4 (Fig. 1c). Cys73 and the “semi-conserved” region will be further discussed later.

### Structural comparison of *Gs*FPPase with mesophilic FPPases

Many studies have been conducted to elucidate the factor(s) that determine thermostability of an enzyme, and the often used method is based on comparison of thermophilic enzymes and their mesophilic homologs. Because prenyltransferases can be divided into prokaryotic and eukaryotic families (Chen et al. 1994), comparison of *Gs*FPPase with both prokaryotic and eukaryotic family members might give some clues about the determinant(s) of its thermostability.

The structure of *Gs*FPPase was compared with *Thermus thermophilus* geranylgeranyl diphosphate synthase (*Tt*GGPPase), human FPPase, and avian FPPase. Human and avian FPPases were selected as representatives of mesophilic FPPases from the eukaryotic family. *Tt*GGPPase was chosen because it is the only thermophilic prenyltransferase from the prokaryotic family whose crystal structure has been solved and is relatively similar to FPPase structure.

**Fig. 2:**
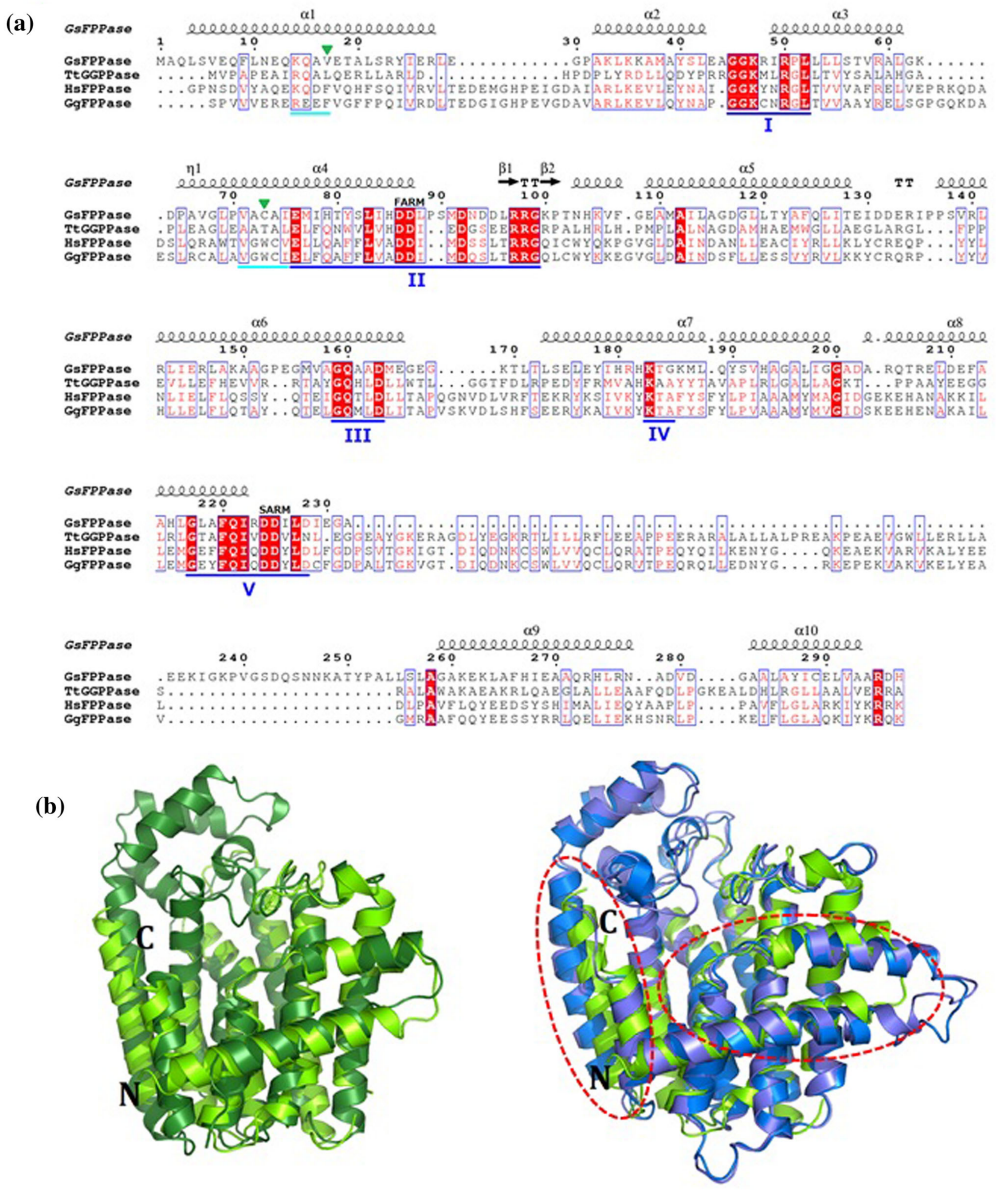
Structural comparison of *Gs*FPPase to its homologues from other species. **a** Multiple sequence alignment of *Gs*FPPase with three selected prenyl diphosphate synthases. Five conserved regions, I–V, are underlined in blue. Semi-conserved regions are *underlined* in *cyan*. Two residues affecting atomic packing (e.g., Val17 and Cys73 in the case of *Gs*FPPase) are indicated by *green inverted triangles*. The alignment was created using ESPript (Robert and Gouet 2014). (*Gs*FPPase, *Geobacillus stearothermophilus* FPP synthase (Uniprot ID: Q08291); *Tt*GGPPase, *Thermus thermophilus* GGPP synthase (Q5SMD0); *Hs*FPPase, *Homo sapiens* FPP synthase (P14324); *Gg*FPPase, *Gallus gallus* FPP synthase (P08836)). **b** Superimposition between *Gs*FPPase and three selected prenyl diphosphate synthases. *Gs*FPPase, *Tt*GGPPase, human FPPase, and avian FPPase are colored in *light green*, *dark green*, *blue*, and *light purple*, respectively

**Fig. 3:**
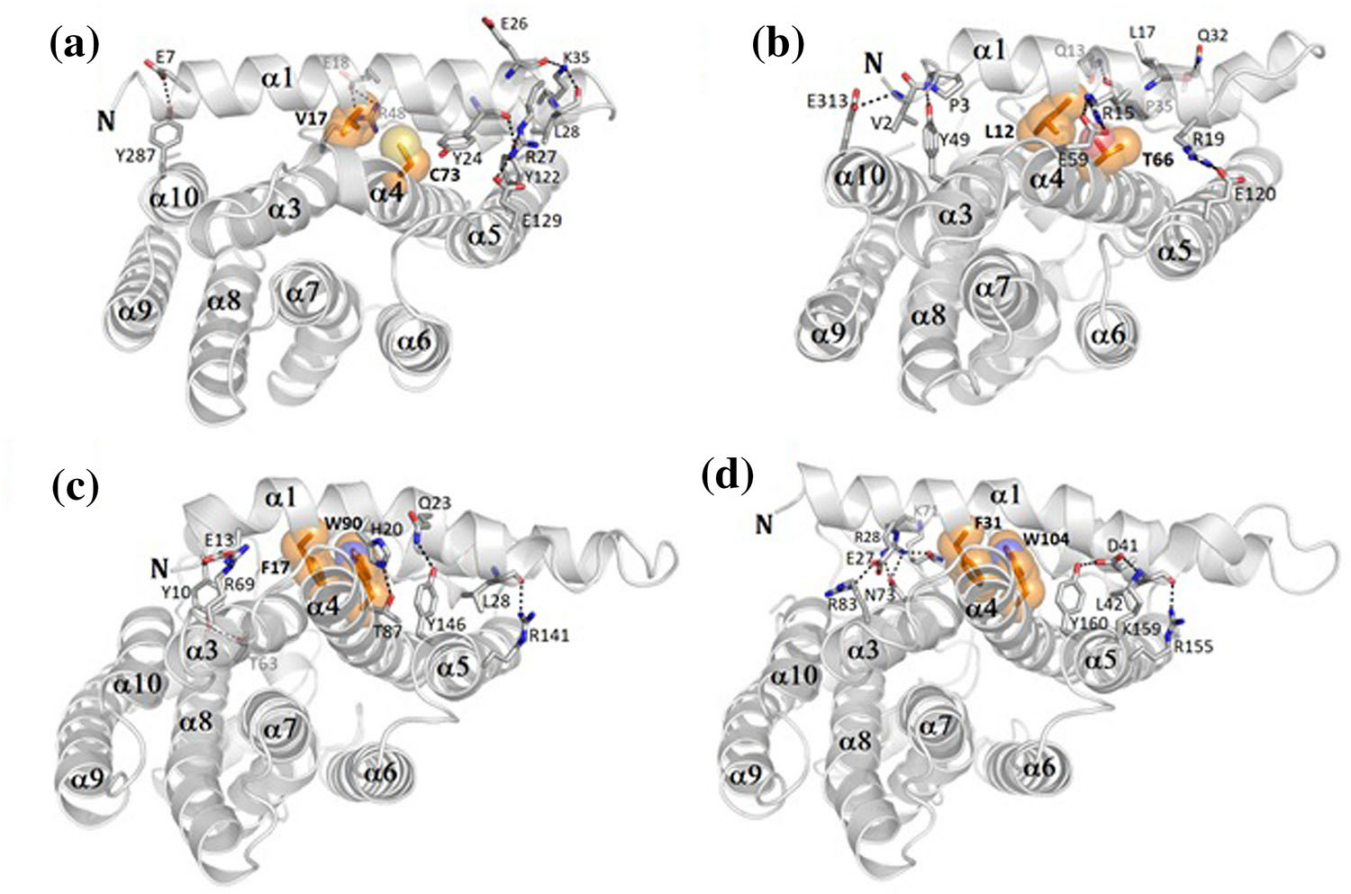
Structural surroundings of semi-conserved regions. Two important residues which affect the atomic packing of helices α1 and α4 are shown in stick and sphere models (*orange*) and residues involved in the interactions are shown as stick models and labeled. Hydrogen bonds are indicated by* dashed lines* (*black*). **a** Semi-conserved regions of *Gs*FPPase. **b** Semi-conserved regions of *Tt*GGPPase. **c** Semi-conserved regions of human FPPase. **d** Semi-conserved regions of avian FPPase

Structure-based multiple sequence alignment of the four selected enzymes showed that these enzymes share five common conserved regions (I–V) of the prenyltransferase family. Two sequences which appear to be semi-conserved regions were also spotted in helices α1 and α4, with one of the regions located before region II (Figs. 1c, 2a). We called these regions “semi-conserved” because residues in these regions are similar between intra-family members but differ for inter-family members. For the eukaryotic families, the semi-conserved region before region II contains a GWC (Glycine-Tryptophan-Cysteine) motif. On the other hand, the corresponding position in the eukaryotic families contains an AXA (Alanine-Unknown-Alanine) motif. Superimposition of the structure of *Gs*FPPase with three other selected prenyltransferases indicated that the overall folding pattern of *Gs*FPPase is highly similar to the other enzymes, especially to *Tt*GGPPase. However, there are significant differences between *Gs*FPPase and its mesophilic homologs, in terms of the conformations of helices α1, α2, α9, and α10 (indicated with red-dashed lines in Fig. 2b). The differences may be attributed to the differences in semi-conserved regions. Differences in size of amino acids in these regions give rise to differences in the tightness of atomic packing of helices α1 and α4, which also affect the overall packing of the surrounding helices (Fig. 3). It seems that atomic packing of orthogonally positioned helices α1 and α4 is most likely influenced by two residues that are upside down relative to each other on the intersection point of α1 and α4. In the case of the eukaryotic family members, the two residues, which are Phe17 and Trp90 in human FPPase or Phe31 and Trp104 in avian FPPase, tend to be more bulky than in thermophilic homologs from the prokaryotic family. The pi–pi stacking interaction of these two residues cause the mesophilic enzymes to have a better or tighter atomic packing of helices α1 and α4, which then causes the atomic packing of other helices, especially between α1 and α10, to become looser (Fig. 3c, d). On other hand, the two residues that are located in the same position in *Gs*FPPase and *Tt*GGPPase are relatively small, thus the atomic packing of α1 and α4 is relatively loose. However, the overall atomic packing of surrounding helices, e.g. atomic packing between α1 and α10, is tight (Fig. 3a, b). Rigidity of overall atomic packing of the enzyme structure is conceivably the factor that determines stability of thermophilic *Gs*FPPase and *Tt*GGPPase. In *Gs*FPPase, the residues are equivalent to Val17 and Cys73, therefore, Val17 and Cys73 are the residues that affect the thermal stability of *Gs*FPPase. In fact, substitution of Cys73 with Phe caused the enzyme to become sensitive to heat treatment (Koyama et al. 1994).

**Fig. 4:**
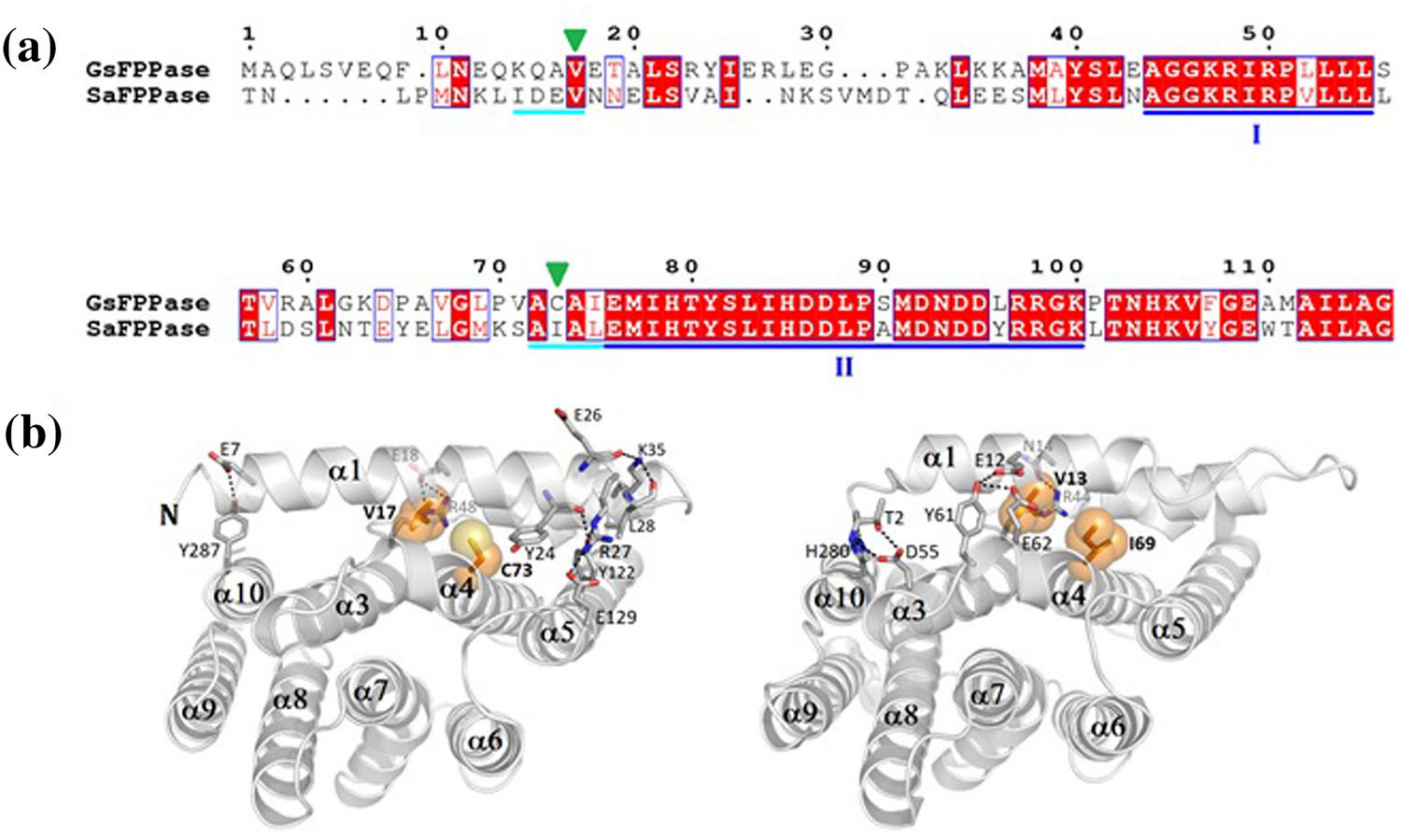
Comparison of *Gs*FPPase to *Sa*FPPase. **a** Sequence alignment of *Gs*FPPase with *Sa*FPPase. Conserved regions I and II are *underlined* in *blue*, and semi-conserved regions are *underlined* in *cyan*. Two residues affecting atomic packing are indicated by *green inverted triangles*. **b** Structural surroundings of semi-conserved regions of *Gs*FPPase (*left*) and *Sa*FPPase (*right*). Two important residues which affect the atomic packing of helices α1 and α4 are shown by *sphere models* (*orange*)

We also compared *Gs*FPPase with FPPase from mesophilic bacteria *Staphylococcus aureus* (*Sa*FPPase). Although *Sa*FPPase shows a high degree of sequence identity (46%) to *Gs*FPPase, the two enzymes are from two different bacteria with distinct thermal properties (Donk 1920; Hughes and Hurst 1980). The remarkable difference between these two enzymes is the size of amino acid residue located on the 13th position before FARM (Fig. 4). Ile69 of *Sa*FPPase is more bulky than Cys73 of *Gs*FPPase, which indicates that size of the residue in this position is one of the possible structural features contributing to thermostability of FPPases.

### Relation of Cys73 and the rigidity of *Gs*FPPase structure

**Fig. 5:**
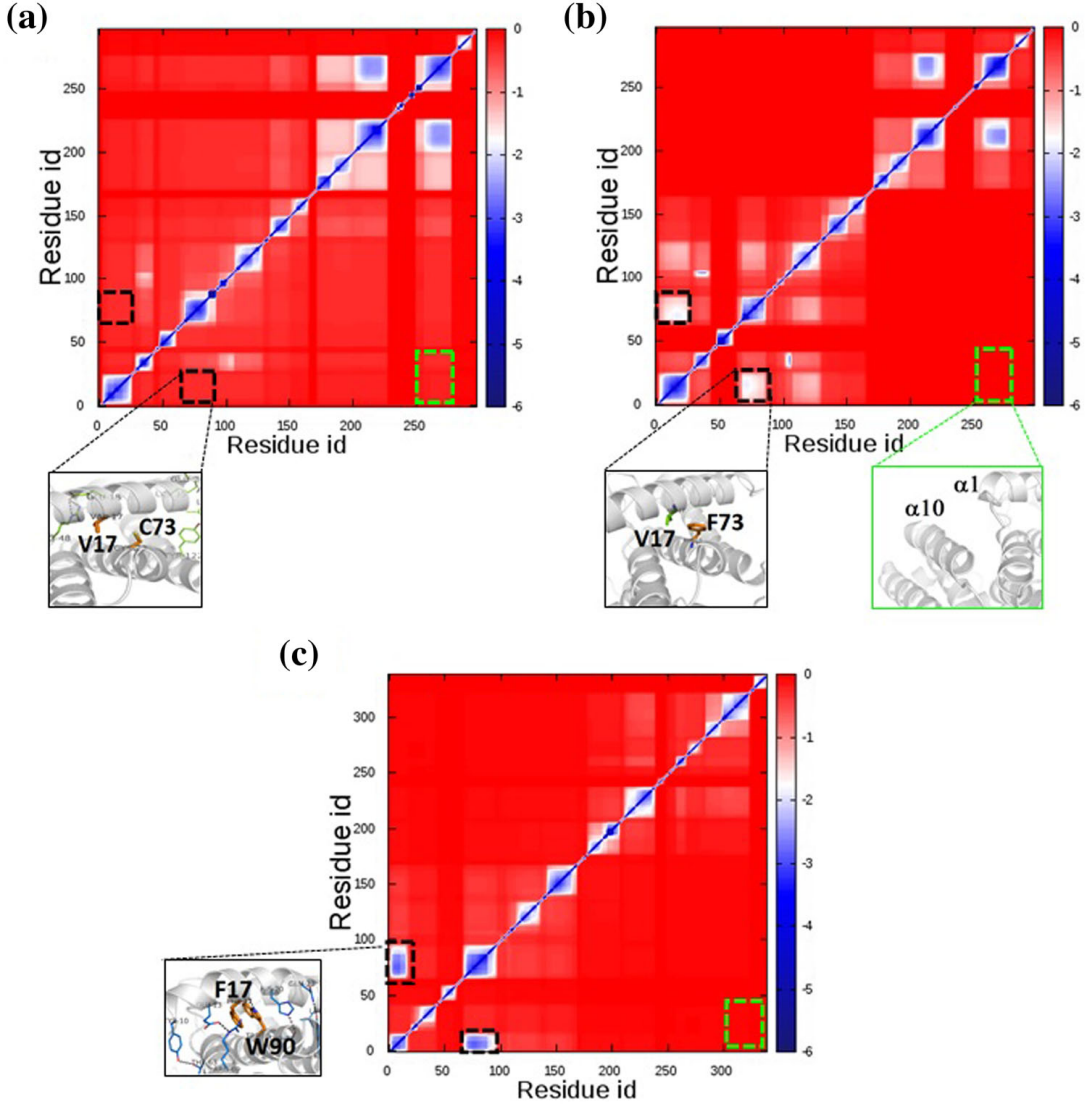
Stability maps of enzymes from the dataset. Relative strengths of rigidity contacts are color-coded: pairs of residues where no or only a weak rigid contact exists are shown in *red*, and pairs of residues with strong rigid contacts are shown in *blue*. Rigid contacts of semi-conserved regions are indicated with *black dashed lines*. Rigid contacts between α1 and α10 are indicated with *green dashed lines*. **a** Stability map of wild-type *Gs*FPPase. **b** Stability map of point-mutated *Gs*FPPase C73F. **c** Stability map of human FPPase

We used Constraint Network Analysis (CNA) to investigate the relationship between Cys73 and the rigidity of *Gs*FPPase structure. The dataset used in this study contains human FPPase (PDB ID: 4XQT), wild-type *Gs*FPPase (PDB ID: 5AYP) and point-mutated *Gs*FPPase (C73F) structures, with all of the structures in the open form (in the absence of a ligand). The calculated *T*_p_ and experimental *T*_m_ of enzymes from the dataset are given in Table 2. Unfortunately, *T*_m_ is not available for C73F, so we used the temperature in which C73F lost most of its enzymatic activity. From Table 2, we see that the calculated *T*_p_ is in good agreement with the experiment. Both of the results showed that the thermostability of C73F is lower than the wild-type of *Gs*FPPase, but higher when compared with human FPPase. To characterize the microscopic factors of these features, we further analyzed the stability map of each enzyme. The stability map indicated rigid contacts between two residues that belong to the same rigid cluster. Examining the stability maps (Fig. 5) reveals that the pattern of rigidity contacts of C73F becomes more similar to that of human FPPase, although C73F still maintains the basic pattern of rigidity contacts of *Gs*FPPase. When Cys73 is replaced with Phe, the strength of rigidity contacts between amino acid 73 with Val17 and with its surrounding residues increases (indicated with black-dashed lines in Fig. 5b). On the other hand, strength of rigidity contacts of remaining residues in C73F decreases, which also means that the thermostability of C73F decreases, compared with wild-type *Gs*FPPase. These simulation results support our hypothesis that replacement of Cys73 with a more bulky amino acid tightens the atomic packing of helices α1 and α4, which then loosens the atomic packing of the remaining helices (e.g. atomic packing between α1 and α10).

**Table 2:** Computed phase transition temperature (*T*_p_) values versus experimental melting point temperature (*T*_m_) values

Enzyme	Phase transition point *T*_p_ (°C)	Melting point *T*_m_ (°C)
Wild-type *Gs*FPPase	69.6	72
C73F	53.7	55^a^
Human FPPase	44.7	50^b^

^a^C73F lost its enzymatic activity after heat treatment at 55 °C for 30 min (Koyama et al. 1994)

^b^ *T*_m_ value was taken from reference (Liu et al. 2014)

To the best of our knowledge, this is the first work that shows that prenyltransferases have “semi-conserved” regions that are crucial for structural stability, and shows how these regions affect the thermostability of the enzyme. Knowledge about thermostability factors of FPPase will open the way for protein engineering to increase its thermostability, and thus widen the industrial applications of FPPase in the future.
